# Impact of the EARL harmonization program on automatic delineation of metabolic active tumour volumes (MATVs)

**DOI:** 10.1186/s13550-017-0279-y

**Published:** 2017-03-31

**Authors:** Charline Lasnon, Blandine Enilorac, Hosni Popotte, Nicolas Aide

**Affiliations:** 1 Nuclear Medicine Department, François Baclesse Cancer Centre, Caen, France; 2 INSERM U1086 « ANTICIPE », BioTICLA, François Baclesse Cancer Centre, Caen, France; 3 Nuclear Medicine Department, University Hospital, Avenue Côte de Nacre, 14000 Caen, France; 4 Radiation Oncology, François Baclesse Cancer Centre, Caen, France; 5 Normandie University, Caen, France

**Keywords:** FDG, PET, MATV, Harmonization, EARL accreditation program

## Abstract

**Background:**

The clinical validation of the EARL harmonization program for standardised uptake value (SUV) metrics is well documented; however, its potential for defining metabolic active tumour volume (MATV) has not yet been investigated. We aimed to compare delineation of MATV on images reconstructed using conventional ordered subset expectation maximisation (OSEM) with those reconstructed using point spread function modelling (PSF-reconstructed images), and either optimised for diagnostic potential (PSF) or filtered to meet the EANM/EARL harmonising standards (PSF_7_).

**Methods:**

Images from 18 stage IIIA-IIIB lung cancer patients were reconstructed using all the three methods. MATVs were then delineated using both a 40% isocontour and a gradient-based method. MATVs were compared by means of Bland–Altman analyses, and Dice coefficients and concordance indices based on the unions and intersections between each pair of reconstructions (PSF vs OSEM, PSF_7_ vs PSF and PSF_7_ vs OSEM).

**Results:**

Using the 40% isocontour method and taking the MATVs delineated on OSEM images as a reference standard, the use of PSF_7_ images led to significantly higher Dice coefficients (median value = 0.96 vs 0.77; *P* < 0.0001) and concordance indices (median value = 0.92 vs 0.64; *P* < 0.0001) than those obtained using PSF images.

The gradient-based methodology was less sensitive to reconstruction variability than the 40% isocontour method; Dice coefficients and concordance indices were superior to 0.8 for both PSF reconstruction methods. However, the use of PSF_7_ images led to narrower interquartile ranges and significantly higher Dice coefficients (median value = 0.96 vs 0.94; *P* = 0.01) and concordance indices (median value = 0.89 vs 0.85; *P* = 0.003) than those obtained with PSF images.

**Conclusion:**

This study demonstrates that automatic contouring of lung tumours on EARL-compliant PSF images using the widely adopted automatic isocontour methodology is an accurate means of overcoming reconstruction variability in MATV delineation. Although gradient-based methodology appears to be less sensitive to reconstruction variability, the use of EARL-compliant PSF images significantly improved the Dice coefficients and concordance indices, demonstrating the importance of harmonised-images, even when more advanced contouring algorithms are used.

**Electronic supplementary material:**

The online version of this article (doi:10.1186/s13550-017-0279-y) contains supplementary material, which is available to authorized users.

## Background

**Fig. 1:**
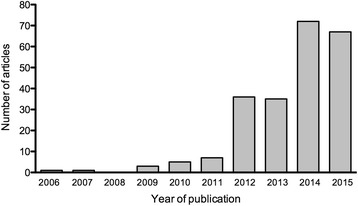
Numbers of articles related to MATV as a function of the year of publication. Publications were identified using a MEDLINE search with the following enquiry: (“MATV” or “MTV”) and “PET”. Only human studies were included

Although standard metrics such as standardised uptake values (either SUV_max_ or SUV_peak_) are widely used as prognostic tools or for monitoring of therapy in cancer treatment [1], metabolically active tumour volume (MATV) has recently been receiving a lot of interest as a pretreatment prognostic tool for various types of cancer [2–5]. Delineation of MATV is also useful for radiotherapy planning in various types of cancer, including non-small cell lung cancer (NSCLC) [6]. This growing interest in MATV is illustrated in Fig. 1, which shows the number of articles using MATV published over the past 10 years. The impact of PET imaging parameters and automatic tumour delineation in radiotherapy planning has been well documented [7–9] and has indicated a requirement for improved delineation methodologies. Recent studies in non-Hodgkin lymphoma (NHL) have shown high MATV to be predictive of overall survival [10]; although, widely disparate cut-off values have been used, which have fuelled the ongoing discussion on the need to standardise the quality of PET images and delineation methods.

Harmonization programs, such as the EANM/EARL (European Association of Nuclear Medicine/EANM Research Ltd) accreditation program [11], are designed to harmonise data acquisition, processing, and analysis to facilitate comparisons of PET quantitative values within multicentre trials, or in sites equipped with multiple PET/CT scanners, regardless of the PET/CT system used. Given that centres running PET systems with advanced reconstruction algorithms often wish to use them with parameters chosen to achieve optimal lesion detection, EARL-accredited centres tend to use two-PET datasets: one for optimal lesion detection and image interpretation and another filtered for harmonised quantification [12].

The EARL program has been well validated for standard SUV metrics [12–15], but clinical validation of this harmonization program for MATV delineation is still lacking. This study examined MATVs delineated in stage IIIA-IIIB lung cancer patients, with the aim of comparing MATVs in PSF-reconstructed images optimised for diagnosis (PSF), PSF-reconstructed images with a filter chosen to meet harmonising standards (PSF_7_), and EARL-compliant images reconstructed using ordered subset expectation maximisation (OSEM). Stage III NSCLCs were chosen, as these stages are typically treated by radiation therapy or radio-chemotherapy, and many centres use FDG PET MATV delineation to optimise tumour targeting. MATVs were compared not only in terms of absolute and relative values but also using a concordance measure, which gives a representative geometrical description of changes in MATV, combining both volume and positional differences [16].

## Methods

### Patient selection

Eighteen consecutive biopsy-proven stage IIIA-IIIB lung cancer patients who had been scanned for staging purposes were included in this retrospective study. This study was approved by the local ethics committee (Ref A12-D24-VOL13, *Comité de protection des personnes Nord-Ouest III*), and the requirement for informed consent was waived.

### PET/CT examinations

Patients who had fasted for 6 h previous to the examination were injected with ^18^F-FDG after 15-min of rest in a warm room (mean injected dose ±SD = 3.89 ± 0.44 MBq/Kg). All PET imaging studies were performed 60 ± 5 min post injection, on a Biograph TrueV system (Siemens Medical Solutions, Erlangen, Germany), with a 6-slice spiral CT component, according to the EANM guidelines [17].

A free-breathing CT acquisition was performed first, using the following parameters: 60 mAs, 130 kVp, pitch 1, and 6 × 2-mm collimation. The PET emission acquisition was then subsequently performed in a 3-D mode. Patients were scanned from the skull base to the mid-thighs, with time per bed acquisitions of 160 and 220 s for normal weight (BMI ≤25 kg/m^2^) and overweight patients (BMI >25 kg/m^2^), respectively.

### PET reconstruction

The Biograph TrueV system is equipped with PSF reconstruction (HD; TrueX, Siemens Medical Solutions) but has no time of flight capability.

The standard reconstruction used in our department was a PSF reconstruction algorithm (HD; TrueX, Siemens Medical Solutions; 3 iterations and 21 subsets) without filtering. We did not use any post filtering as modelling the PSF during the iterative reconstruction introduces correlations between neighbouring voxels in a manner similar to smoothing filters and thus has been shown to achieve maximal performance with little to no filtering [18]. Raw data were also reconstructed with an OSEM reconstruction algorithm (4 iterations and 8 subsets), and a PSF reconstruction algorithm (3 iterations and 21 subsets) incorporating a 7-mm Gaussian filter (PSF_7_). As shown in a previous study [12], this latter reconstruction leads to protocol-specific images with NEMA NU-2 phantom-based filtering that meet EANM 1.0 quantitative harmonisation standards. The OSEM reconstruction parameters also met the EANM requirements on activity recovery.

The matrix size for all reconstructions was 168 × 168 voxels, resulting in isotropic voxels of 4.07 × 4.07 × 4.07 mm. Scatter and CT attenuation corrections were also applied.

### PET tumour delineation

PET images were contoured by two experienced PET readers using MIM image-contouring tools (MIM-5.6, MIM Software Inc, Cleveland, OH). Two different contouring methods were performed, a 40% of SUV_max_ thresholding technique and a gradient-based technique involving the PET edge contouring tool [19, 20]. The procedures focused only on the primary tumour and did not include involved node(s), except in cases of bulky disease, where tumoural and nodal uptake could not be separated.

### Comparison of tumour volumes and statistical analysis

**Fig. 2:**
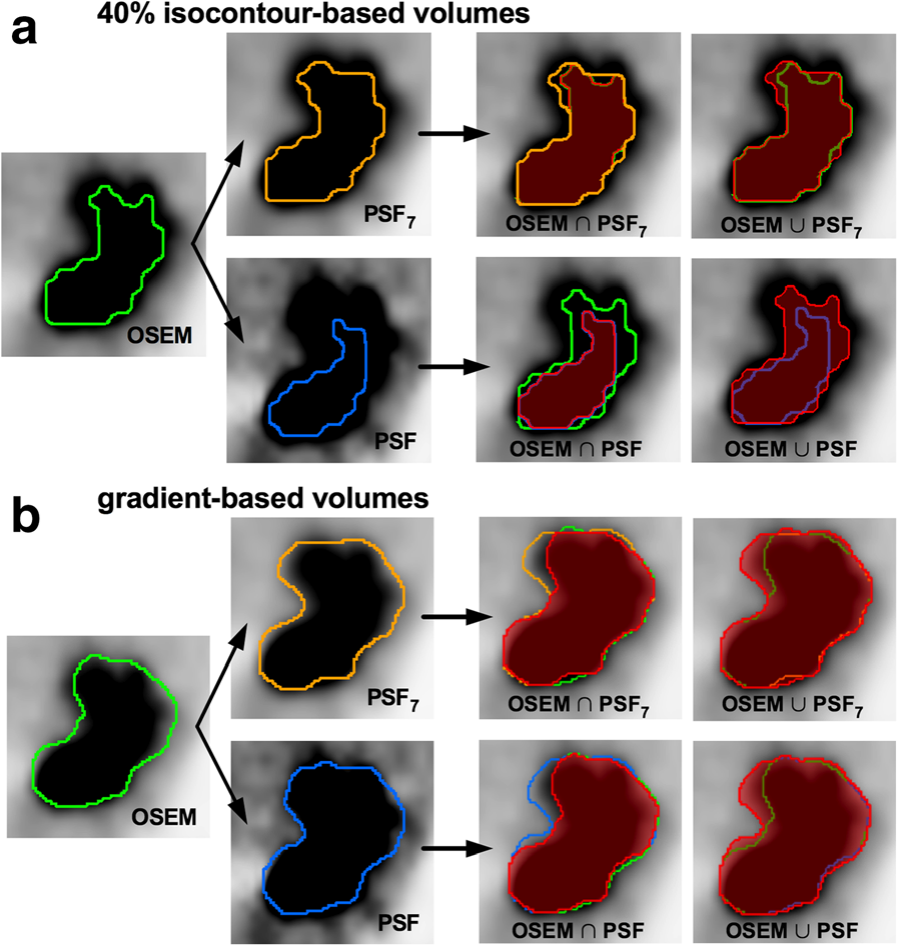
A representative example of automatic delineation of a tumour. MATVs derived from OSEM, PSF, and PSF_7_ reconstructions, as well as their unions and intersections, are shown on axial slices from a 61-year-old patient presenting with a stage IIIA squamous cell lung cancer. Panels **a** and **b** display 40% isocontour-based volumes and gradient-based volumes, respectively

MATVs were compared by determining the union and the intersection between each pair of reconstruction methods (PSF vs OSEM and PSF_7_ vs OSEM), and then computing the Dice coefficients and concordance indices as follows:

$$ \mathrm{Dice}'\mathrm{s}\ \mathrm{coefficients} = \frac{2\left(\mathrm{MATV}1\ \cap \mathrm{MATV}2\right)}{\mathrm{MATV}1 + \mathrm{MATV}2} $$

$$ \mathrm{Concordance}\mathrm{indices}=\frac{\mathrm{MATV}1\kern0.5em \cap \mathrm{MATV}2}{\mathrm{MATV}1\cup \mathrm{MATV}2} $$

where MATV1 and MATV2 are two volumes delineated on different reconstructions for a given tumour and ∪ and ∩ are respectively the union and the intersection between the volumes. Representative volumes and their union and intersection are shown in Fig. 2.

These indices give a representative geometrical description of changes in MATVs, combining both volume and positional changes [16]. Their values vary between 0 where the MATVs are completely disjointed and 1 where the MATVs match perfectly in terms of size, shape, and location.

Quantitative data are presented as mean and standard deviation (SD) or median and interquartile range, as appropriate. Bland–Altman analyses were used to compare MATVs obtained using the three reconstruction methods. The metrics obtained on each of the three sets of PET images were compared globally using Friedman tests with a post hoc Dunn test [21] used to compare each pair of reconstructions (PSF vs OSEM, PSF_7_ vs PSF and PSF_7_ vs OSEM). The Friedman non-parametric test was chosen because not all the quantitative values had a normal distribution, as tested with the Shapiro Wilk normality test.

The Dice coefficients and concordance indices between the OSEM and PSF or PSF_7_ reconstructions were compared using the Wilcoxon test for paired samples. Inter-observer variability was assessed with Lin’s concordance coefficient [11]. Moreover, the whole analysis (comparison of volumes and Dice coefficients and concordance indices) was performed in duplicate with volumes extracted by the two observers. For all statistical tests, a two-tailed *P* value of less than 0.05 was considered statistically significant. Graphs and analyses were performed using Prism (version 5.0f, GraphPad Software, La Jolla, CA).

## Results

### Patient characteristics

Eighteen patients (16 males, 2 females; mean (±SD) age 61 (±11) years) were included. The patient characteristics and their TNM and AJCC stages are listed in Tables 1 and 2, respectively.

**Table 1:** Patient characteristics

Characteristic	
Gender	
Males	16 (88.8)
Females	2 (11.1)
Age (years)	
Range	37–81
Mean (SD)	61 (10.9)
Body habitus (kg/m^2^), *n* (%)	
BMI ≤25	10 (55.6)
BMI >25	8 (44.4)
Histological diagnosis, *n* (%)	
Small cell lung cancer	1 (5.6)
Non-small cell lung cancer	17 (94.4)
Squamous cell carcinoma	10 (55.6)
Adenocarcinoma	5 (27.8)
Large cell lung cancer	2 (11.1)

**Table 2:** TNM stages and AJCC anatomic stage

Patients	TNM	AJCC stage
1	T2N2M0	IIIA
2	T3N1M0	IIIA
3	T2N2M0	IIIA
4	T4N3M0	IIIB
5	T3N1M3	IIIA
6	T4N0M0	IIIA
7	T3N2M0	IIIA
8	T4N2M0	IIIB
9	T4N1M0	IIIA
10	T3N2M0	IIIA
11	T4N0M0	IIIA
12	T4N0M0	IIIA
13	T2N3M0	IIIB
14	T4N2M0	IIIB
15	T3N2M0	IIIA
16	T2N2M0	IIIA
17	T4N1M0	IIIA
18	T1N2M0	IIIA

### Comparison of tumour volumes calculated using the three reconstruction techniques

#### Forty percent isocontour method

**Fig. 3:**
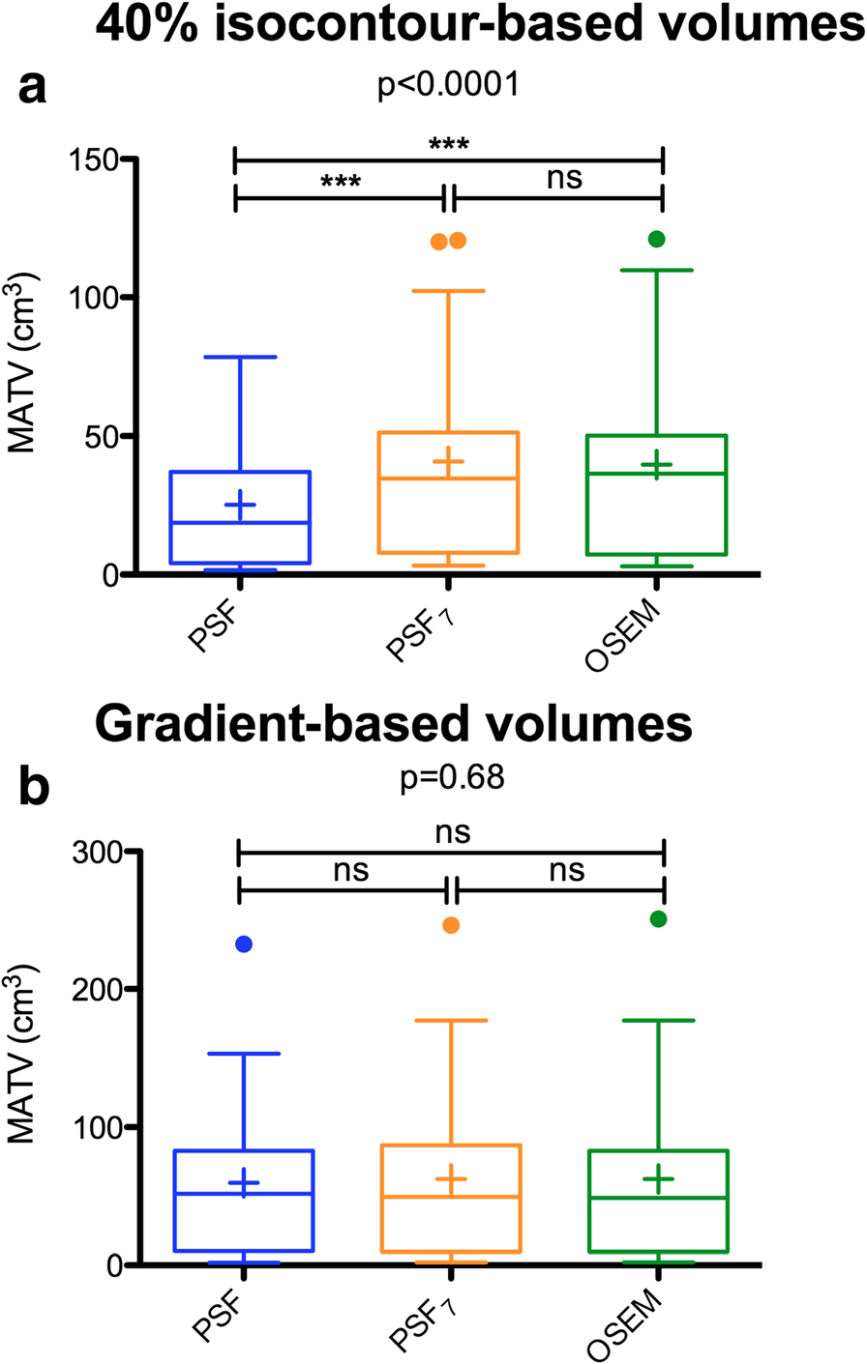
Impact of the EARL harmonization strategy on MATVs defined by the isocontour and gradient-based delineation methods (observer 1). MATVs are shown as *Tukey boxplots* (*lines* displaying the median, 25th, and 75th percentiles; *cross* represents the mean values). Legends for *p* values: ***<0.001; **<0.01; *<0.05. *ns* non significant. **a** 40% isocontour-based volumes. **b** Gradient-based volumes

Tumour delineation on unfiltered PSF images resulted in significantly smaller volumes (median = 18.6 cm^3^, interquartile range 4 to 37) than obtained with the OSEM algorithm (median = 36.4 cm^3^, interquartile range 7.1 to 50.2; *P* < 0.001). The use of EARL-compliant PSF images (PSF_7_) resulted in volumes similar to those obtained with OSEM reconstructions (median = 34.6 cm^3^, interquartile range 7.9 to 51.4; no significant difference; Fig. 3a).

**Fig. 4:**
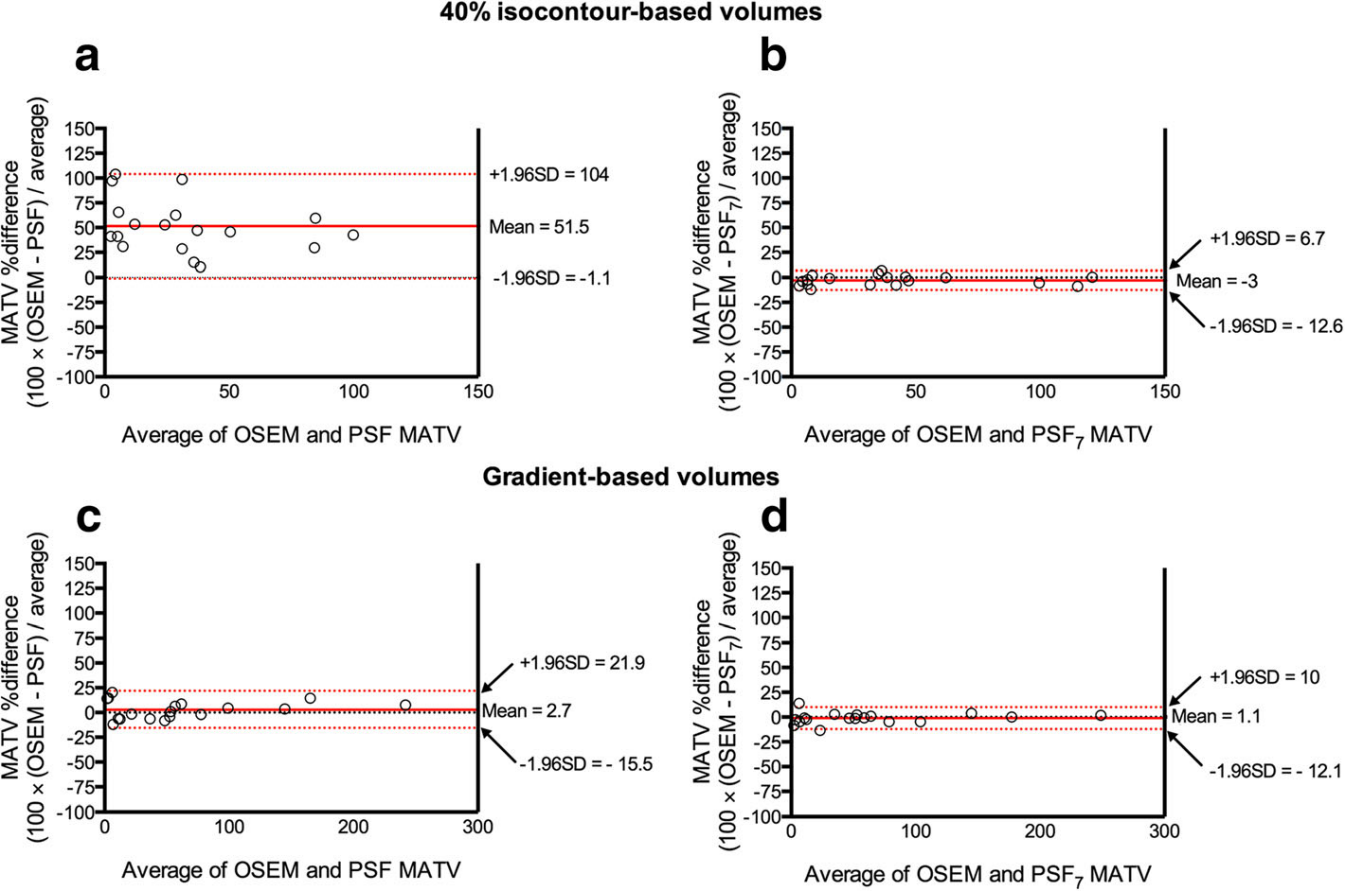
Comparison of MATVs from PSF images optimised for diagnostic potential and EARL-compliant PSF_7_ images (observer 1). Relationships between MATVs extracted from OSEM reconstructions and PSF or PSF_7_ reconstructions, for the 40% isocontour-based methods (**a**, **b**) and gradient-based methods (**c**, **d**), assessed using Bland–Altman plots

The mean percentage difference between unfiltered PSF and OSEM reconstructions for isocontour-based MATVs was 51.5% (95% CI −1.1 to 104; Fig. 4a). After application of the 7-mm Gaussian filter, this difference was reduced to −3% (95% CI −12.6 to 16.7; Fig. 4b).

#### Gradient-based method

When the PET edge tool was utilised, no significant difference was observed in the volumes delineated by the unfiltered PSF (median = 51.3 cm^3^, interquartile range 10.2 to 82.8), OSEM (median = 48.7 cm^3^, interquartile range 9.6 to 82.8), and PSF_7_ (median = 49.3 cm^3^, interquartile range 9.6 to 86.8) reconstructions (Fig. 3b).

The mean percentage difference between the unfiltered PSF and OSEM reconstructions for isocontour-based MATVs was 2.7% (95% CI −15.5 to 21.9; Fig. 4c). After application of the 7-mm Gaussian filter, the mean percentage difference was reduced to 1.1% (95% CI −12.1 to 10; Fig. 4d).

### Concordance between MATVs from unfiltered PSF, OSEM, and PSF EARL-compliant reconstructions

#### Forty percent isocontour method

**Fig. 5:**
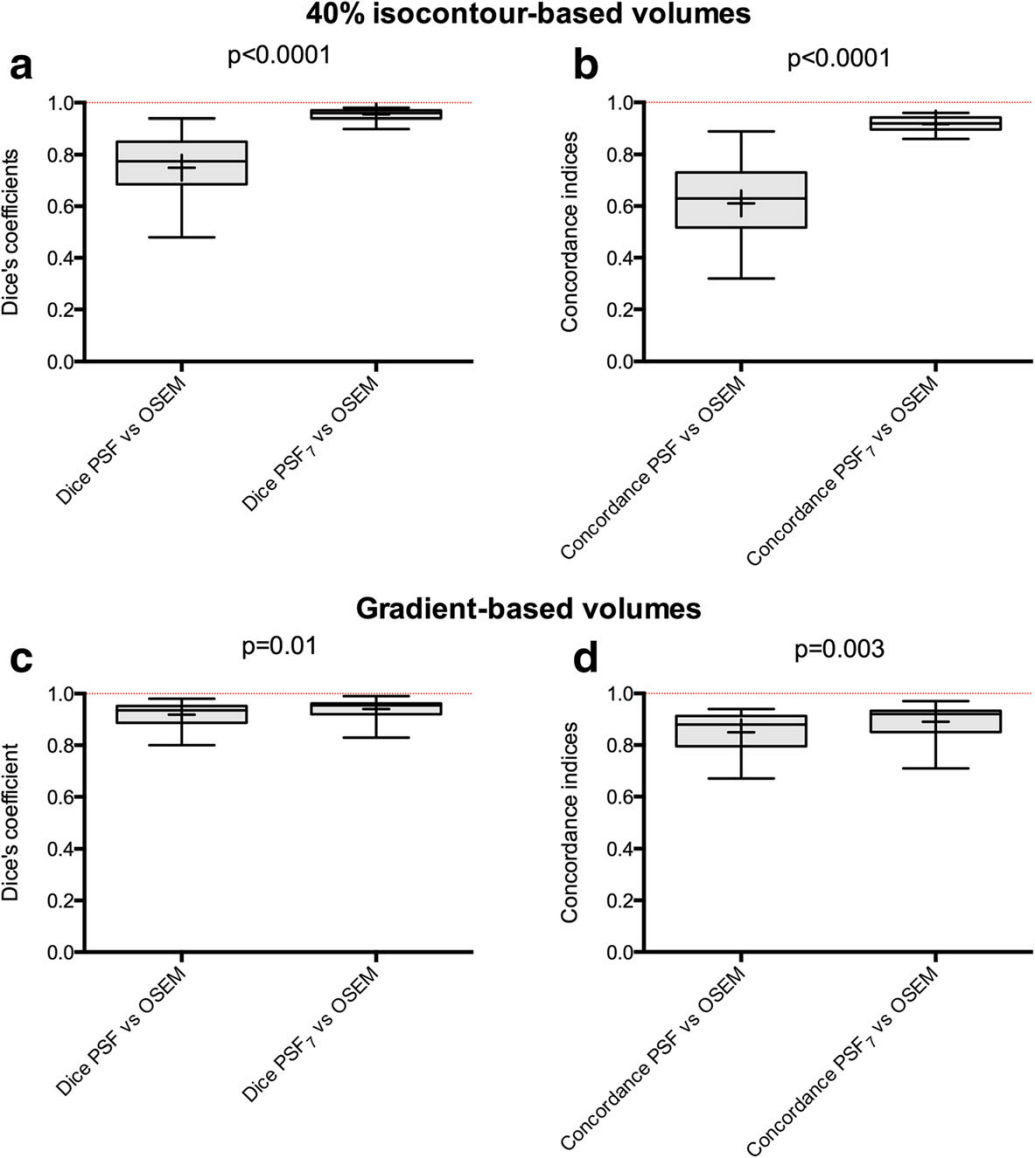
Impact of the EARL harmonization strategy on Dice and concordance indices between MATVs extracted from OSEM images and PSF images (observer 1). The PSF_7_ images were filtered to meet EARL requirements while PSF images were optimised for diagnostic potential. Data are shown as *Tukey boxplots* (*lines* displaying the median, 25th, and 75th percentiles; *crosses* represents the mean values). Dice coefficients and concordance indices are shown for both the isocontour-based method (**a**, **b**) and gradient-based method (**c**, **d**). *ns* not significant

With consideration of MATVs delineated on OSEM images as the reference standard, the use of PSF_7_ images resulted in significantly higher Dice coefficients (median value = 0.96 vs 0.77, *P* < 0.0001; Fig. 5a) and concordance indices (median value = 0.92 vs 0.64, *P* < 0.0001; Fig. 5b) than those obtained with unfiltered PSF images. The interquartile ranges were also narrower when the PSF_7_ images were used.

#### Gradient-based method

In comparison to the OSEM method, the Dice coefficients and concordance indices were superior to 0.8 with either the unfiltered PSF- or PSF_7_-delineated MATVs. Despite this high similarity, the use of PSF_7_ images resulted in significantly higher Dice coefficients (median value = 0.96 vs 0.94; *P* = 0.01) and concordance indices (median value = 0.89 vs 0.85; *P* = 0.003), and narrower interquartile ranges were observed for PSF_7_ images (Fig. 5c, d).

**Fig. 6:**
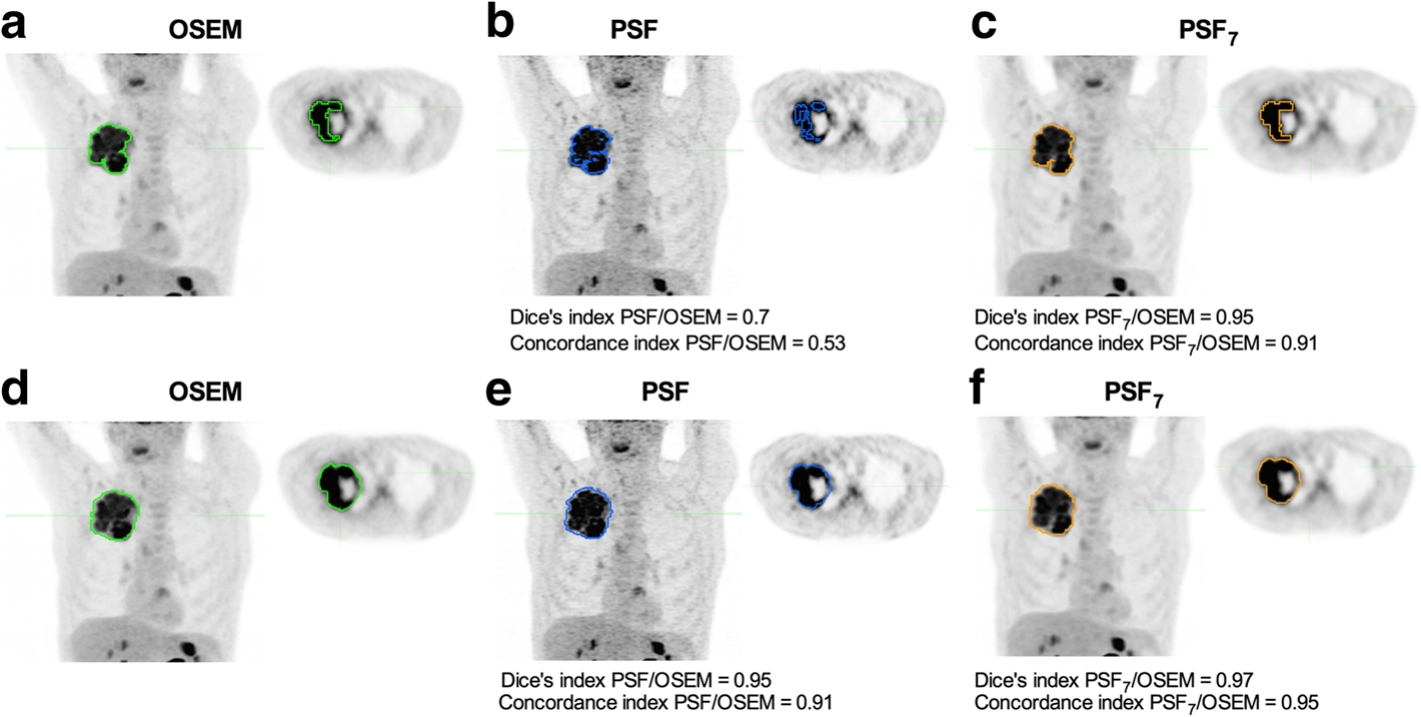
Representative images of isocontour- and gradient-based automatic contouring of PSF images optimised for diagnostic and EARL-compliant purposes. Maximum intensity projections and transverse slices at the level of a necrotic tumour in the right upper lobe of the lung are shown for OSEM, unfiltered PSF images, and PSF images filtered to meet EARL requirements (PSF_7_). Dice and concordance indices are given for each contouring method, using the MATV extracted from the OSEM images as a reference standard

Representative images of the isocontour- and gradient-based tumour delineations using all the three reconstruction methods are shown in Fig. 6.

### Inter-observer variability for the 40% isocontour and gradient-based methods

There was an almost perfect inter-observer agreement between each pair of volumes assessed by both observers, with Lin concordance coefficient greater than 0.99 in all cases (Additional file 1: Figure S1).

Regarding the comparison of tumour volumes calculated using the three reconstruction techniques, similar trends were found for both observers, except when comparing PSF and OSEM volumes delineated with the gradient-based method: for observer 2, a statistically significant difference was found between OSEM and PSF volumes (Additional file 2: Figure S2).

When it comes to the concordance between MATVs from unfiltered PSF, OSEM, and PSF EARL-compliant reconstructions, similar results were obtained (Additional file 3: Figure S3). Using the 40% isocontour method and taking the MATVs delineated on OSEM images as a reference standard, the use of PSF_7_ images led to significantly higher Dice coefficients (median value = 0.96 vs 0.75, *P* = 0.0002) and concordance indices (median value = 0.93 vs 0.61, *P* = 0.0002) than those obtained using PSF images. The gradient-based methodology was also found less sensitive to reconstruction variability than the 40% isocontour method. Dice coefficients and concordance indices were superior to 0.8 for both PSF reconstruction methods. The use of PSF_7_ images led to narrower interquartile ranges and significantly higher Dice coefficients (median value = 0.95 vs 0.94; *P* = 0.01) and concordance indices (median value = 0.91 vs 0.89; *P* = 0.02) than those obtained with PSF images.

## Discussion

This study demonstrates that automatic contouring of lung tumours on EARL-compliant PSF images using a widely adopted automatic isocontour methodology is an accurate means of overcoming reconstruction variability in MATV delineation. With OSEM used as a reference, this harmonization strategy led to concordance indices greater than 0.9, with very narrow confidence intervals. This supports the use of EARL-compliant images in multicentre studies, where MATVs extracted from 18F-FDG PET are used for tumour targeting or as a prognostic tool (for example, using the median value of pooled data as a cut-off value). EARL-compliant images could also be used in clinical routine in centres running more than one PET system, a situation that is more frequently being encountered.

The gradient-based methodology appears less sensitive to reconstruction variability, with median values for Dice coefficients and concordance indices between the MATVs delineated on OSEM and PSF images greater than 0.8. However, the use of EARL-compliant PSF images significantly improved these indices, demonstrating the value of harmonised-images, even when more advanced contouring algorithms such as gradient-based contouring are utilised.

In this study, we focused on PSF reconstruction, which implements the detector response function. At the edges of the field of view (FOV), photons are likely to strike crystals at an angle and, as the depth-of-interaction is not known, they may travel through another crystal before they light up. This phenomenon leads to incorrect lines of response, especially at the edge of the FOV; therefore, resolution is not uniform throughout the FOV. The aim of PSF reconstruction is to minimise this effect, thus decreasing partial volume effects, which in turn decreases the spill-in and spill-over within and around a tumour lesion and improves image contrast. In line with these properties of PSF reconstruction, contouring around lung tumours with the isocontour methodology on PSF images led to significantly smaller volumes than obtained from OSEM images. PSF modelling is available from the major PET vendors [22–24]. Though the improvement of spatial resolution may vary depending on the PET system, as well as corrections for the Gibbs artefact [25–27] impacting on quantitation for small lesions, PSF reconstruction consistently increases SUV metrics compared to standard OSEM reconstruction. Therefore, we feel that results similar to those reported in the present study would be obtained with other systems when using the isocontour method.

The present study did not explore other methods for improving tumour delineation on PET/CT images, such as advanced contouring methodologies like contrast-orientated isocontour [28, 29] or the FLAB algorithm [30]. The parameters accounting for the respective weight of tumour and background uptake in the choice of the optimal threshold used in the contrast-orientated isocontour are known to be specific to the system used [29]. We therefore assume that EARL-compliant images would be useful for contouring of tumours with this algorithm in a multicentre setting.

With regards to the use of MATV for radiotherapy planning, our study focused on the initial and crucial step of automatic tumour contouring on PET images. We used automatic contouring so that no other confounding factor such as inter-observer variability [6] could affect the MATVs. As reported in a recent consensus paper from the IAEA [31], the final gross tumour volume used for tumour targeting will also depend on other diagnostic modalities and the use of margin and consensus reading between PET readers and radiation oncologists.

## Conclusion

This study shows that automatic contouring of lung tumours on EARL-compliant PSF images using the widely adopted automatic isocontour methodology is an accurate means to overcome reconstruction variability in MATV delineation. Although the gradient-based methodology appears to be less sensitive to reconstruction variability, the use of EARL-compliant PSF images significantly improved the Dice coefficient and concordance indices, suggesting that the use of harmonised-images is still important, even with more advanced contouring algorithms.

## Additional files

Additional file 1: Figure S1. Inter-observer concordance for volume delineation. Relationships between MATVs extracted from OSEM reconstructions and PSF or PSF_7_ reconstructions for observers were compared using the Lin concordance coefficient (ρ_c_) for the 40% isocontour (a) and gradient-based (b) methods. (TIFF 8917 kb)

Additional file 2: Figure S2. Impact of the EARL harmonization strategy on MATVs defined by the isocontour and gradient-based delineation methods (observer 2). MATVs are shown as Tukey boxplots (lines displaying the median, 25th and 75th percentiles; cross represents the mean values). Legends for *p* values: ***<0.001; **<0.01; *<0.05. ns, not significant. (TIFF 17693 kb)

Additional file 3: Figure S3. Impact of the EARL harmonization strategy on Dice and concordance indices between MATVs extracted from OSEM images and PSF images (observer 2). The PSF_7_ images were filtered to meet EARL requirements while PSF images were optimised for diagnostic potential. Data are shown as Tukey boxplots (lines displaying the median, 25th, and 75th percentiles; crosses represents the mean values). Dice coefficients and concordance indices are shown for both the isocontour method (a and b) and gradient-based method (c and d). ns, not significant. (TIFF 17434 kb)

## Abbreviations

EANM: European Association of Nuclear Medicine
MATV: Metabolic active tumour volume
NHL: Non-Hodgkin lymphoma
NSCLC: Non-small cell lung cancer
OSEM: Ordered subset expectation maximisation
PET/CT: Positron emission tomography/computed tomography
PSF: Point-spread function
SUV: Standardised uptake value

## References

[1] Shang J, Ling X, Zhang L, Tang Y, Xiao Z, Cheng Y (2016). Comparison of RECIST, EORTC criteria and PERCIST for evaluation of early response to chemotherapy in patients with non-small-cell lung cancer. Eur J Nucl Med Mol Imaging.

[2] Bazan JG, Duan F, Snyder BS, Horng D, Graves EE, Siegel BA (2017). Metabolic tumor volume predicts overall survival and local control in patients with stage III non-small cell lung cancer treated in ACRIN 6668/RTOG 0235. Eur J Nucl Med Mol Imaging.

[3] Bazan JG, Koong AC, Kapp DS, Quon A, Graves EE, Loo BW (2013). Metabolic tumor volume predicts disease progression and survival in patients with squamous cell carcinoma of the anal canal. J Nucl Med.

[4] Gauthe M, Richard-Molard M, Fayard J, Alberini JL, Cacheux W, Lievre A (2017). Prognostic impact of tumour burden assessed by metabolic tumour volume on FDG PET/CT in anal canal cancer. Eur J Nucl Med Mol Imaging.

[5] Takeuchi S, Rohren EM, Abdel-Wahab R, Xiao L, Morris JS, Macapinlac HA (2016). Refining prognosis in patients with hepatocellular carcinoma through incorporation of metabolic imaging biomarkers. Eur J Nucl Med Mol Imaging.

[6] van Baardwijk A, Bosmans G, Boersma L, Buijsen J, Wanders S, Hochstenbag M (2007). PET-CT-based auto-contouring in non-small-cell lung cancer correlates with pathology and reduces interobserver variability in the delineation of the primary tumor and involved nodal volumes. Int J Radiat Oncol Biol Phys.

[7] Cheebsumon P, Boellaard R, de Ruysscher D, van Elmpt W, van Baardwijk A, Yaqub M (2012). Assessment of tumour size in PET/CT lung cancer studies: PET- and CT-based methods compared to pathology. EJNMMI Res.

[8] Cheebsumon P, van Velden FH, Yaqub M, Frings V, de Langen AJ, Hoekstra OS (2011). Effects of image characteristics on performance of tumor delineation methods: a test-retest assessment. J Nucl Med.

[9] Cheebsumon P, Yaqub M, van Velden FH, Hoekstra OS, Lammertsma AA, Boellaard R (2011). Impact of [(1)(8)F]FDG PET imaging parameters on automatic tumour delineation: need for improved tumour delineation methodology. Eur J Nucl Med Mol Imaging.

[10] Mikhaeel NG, Smith D, Dunn JT, Phillips M, Moller H, Fields PA (2016). Combination of baseline metabolic tumour volume and early response on PET/CT improves progression-free survival prediction in DLBCL. Eur J Nucl Med Mol Imaging.

[11] Lin LI (1989). A concordance correlation coefficient to evaluate reproducibility. Biometrics.

[12] Lasnon C, Desmonts C, Quak E, Gervais R, Do P, Dubos-Arvis C (2013). Harmonizing SUVs in multicentre trials when using different generation PET systems: prospective validation in non-small cell lung cancer patients. Eur J Nucl Med Mol Imaging.

[13] Lasnon C, Salomon T, Desmonts C, Do P, Oulkhouir Y, Madelaine J (2016). Generating harmonized SUV within the EANM EARL accreditation program: software approach versus EARL-compliant reconstruction. Ann Nucl Med.

[14] Quak E, Le Roux PY, Hofman MS, Robin P, Bourhis D, Callahan J (2015). Harmonizing FDG PET quantification while maintaining optimal lesion detection: prospective multicentre validation in 517 oncology patients. Eur J Nucl Med Mol Imaging.

[15] Quak E, Le Roux PY, Lasnon C, Robin P, Hofman MS, Bourhis D (2016). Does PET SUV harmonization impact PERCIST response classification? Journal of nuclear medicine : official publication. Soc Nucl Med.

[16] Hanna GG, Hounsell AR, O'Sullivan JM (2010). Geometrical analysis of radiotherapy target volume delineation: a systematic review of reported comparison methods. Clin Oncol (R Coll Radiol).

[17] Kuhnert G, Boellaard R, Sterzer S, Kahraman D, Scheffler M, Wolf J (2015). Impact of PET/CT image reconstruction methods and liver uptake normalization strategies on quantitative image analysis. Eur J Nucl Med Mol Imaging.

[18] Kadrmas DJ, Casey ME, Conti M, Jakoby BW, Lois C, Townsend DW (2009). Impact of time-of-flight on PET tumor detection. J Nucl Med.

[19] Callahan J, Kron T, Schneider-Kolsky M, Dunn L, Thompson M, Siva S (2013). Validation of a 4D-PET maximum intensity projection for delineation of an internal target volume. Int J Radiat Oncol Biol Phys.

[20] Geets X, Lee JA, Bol A, Lonneux M, Gregoire V (2007). A gradient-based method for segmenting FDG-PET images: methodology and validation. Eur J Nucl Med Mol Imaging.

[21] Dunn OJ (1964). Multiple comparisons using rank sums. Technometrics.

[22] Bettinardi V, Presotto L, Rapisarda E, Picchio M, Gianolli L, Gilardi MC (2011). Physical performance of the new hybrid PETCT Discovery-690. Med Phys.

[23] Kotasidis FA, Zaidi H (2014). Experimental evaluation and basis function optimization of the spatially variant image-space PSF on the Ingenuity PET/MR scanner. Med Phys.

[24] Panin VY, Kehren F, Michel C, Casey M (2006). Fully 3-D PET reconstruction with system matrix derived from point source measurements. IEEE Trans Med Imaging.

[25] Nuyts J (2014). Unconstrained image reconstruction with resolution modelling does not have a unique solution. EJNMMI Physics.

[26] Rogasch JM, Hofheinz F, Lougovski A, Furth C, Ruf J, Grosser OS (2014). The influence of different signal-to-background ratios on spatial resolution and F18-FDG-PET quantification using point spread function and time-of-flight reconstruction. EJNMMI Physics.

[27] Zeng GL (2011). Gibbs artifact reduction by nonnegativity constraint. J Nucl Med Technol.

[28] Schaefer A, Kremp S, Hellwig D, Rube C, Kirsch CM, Nestle U (2008). A contrast-oriented algorithm for FDG-PET-based delineation of tumour volumes for the radiotherapy of lung cancer: derivation from phantom measurements and validation in patient data. Eur J Nucl Med Mol Imaging.

[29] Schaefer A, Nestle U, Kremp S, Hellwig D, Grgic A, Buchholz HG (2012). Multi-centre calibration of an adaptive thresholding method for PET-based delineation of tumour volumes in radiotherapy planning of lung cancer. Nuklearmedizin Nucl Med.

[30] Hatt M, Cheze Le Rest C, Albarghach N, Pradier O, Visvikis D (2011). PET functional volume delineation: a robustness and repeatability study. Eur J Nucl Med Mol Imaging.

[31] Konert T, Vogel W, MacManus MP, Nestle U, Belderbos J, Gregoire V (2015). PET/CT imaging for target volume delineation in curative intent radiotherapy of non-small cell lung cancer: IAEA consensus report 2014. Radiother Oncol.

