# Supplementary figures and images for: Impact of the EARL harmonization program on automatic delineation of metabolic active tumour volumes (MATVs)
Source: EJNMMI Res. 2017 Mar 31;7:30. doi: 10.1186/s13550-017-0279-y (PMC5374086; doi:10.1186/s13550-017-0279-y)

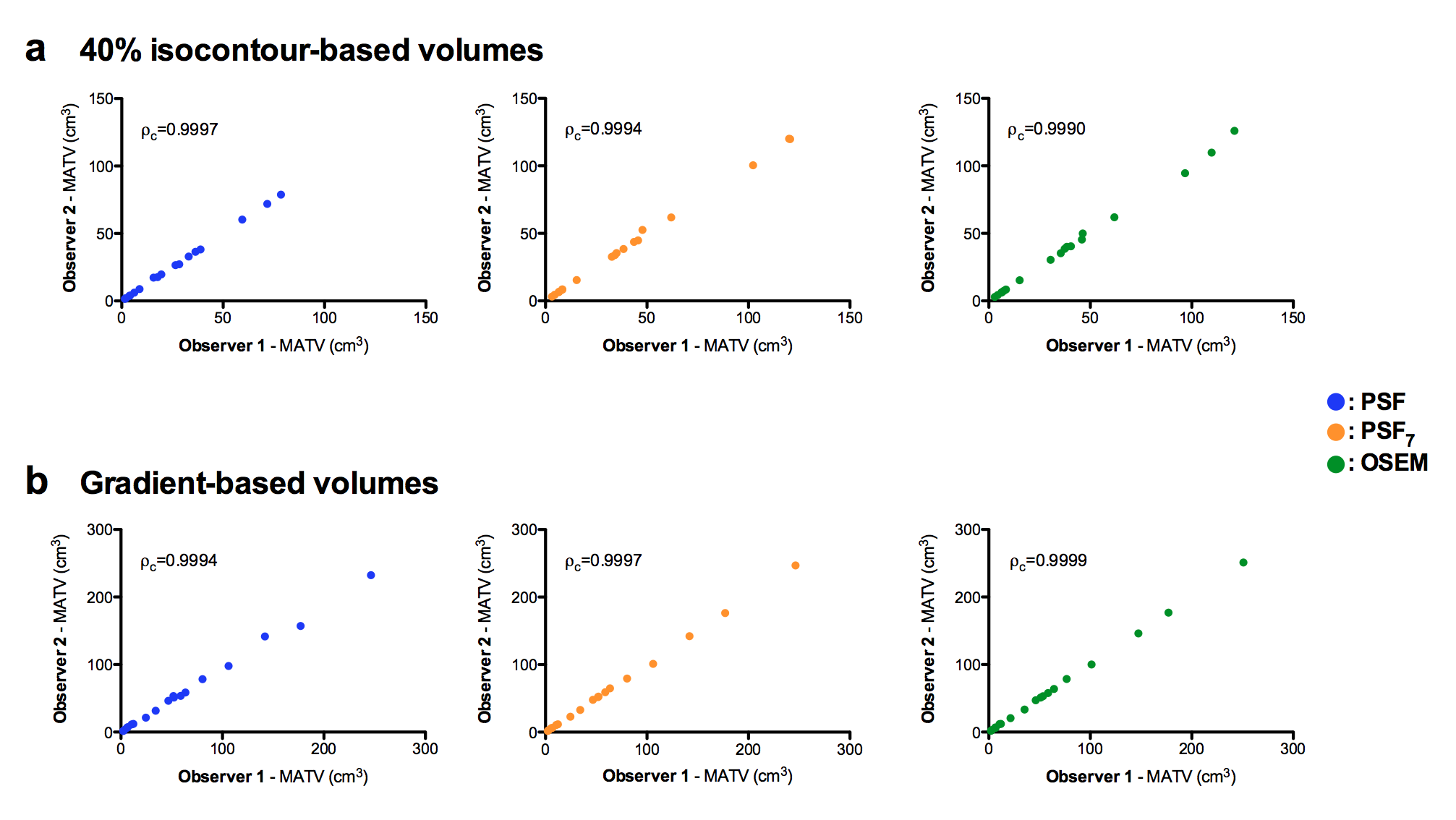

Supplement: Supplementary file 1 — Inter-observer concordance for volume delineation. Relationships between MATVs extracted from OSEM reconstructions and PSF or PSF7 reconstructions for observers were compared using the Lin concordance coefficient (ρc) for the 40% isocontour (a) and gradient-based (b) methods. (TIFF 8917 kb) [file 13550_2017_279_MOESM1_ESM.tiff]

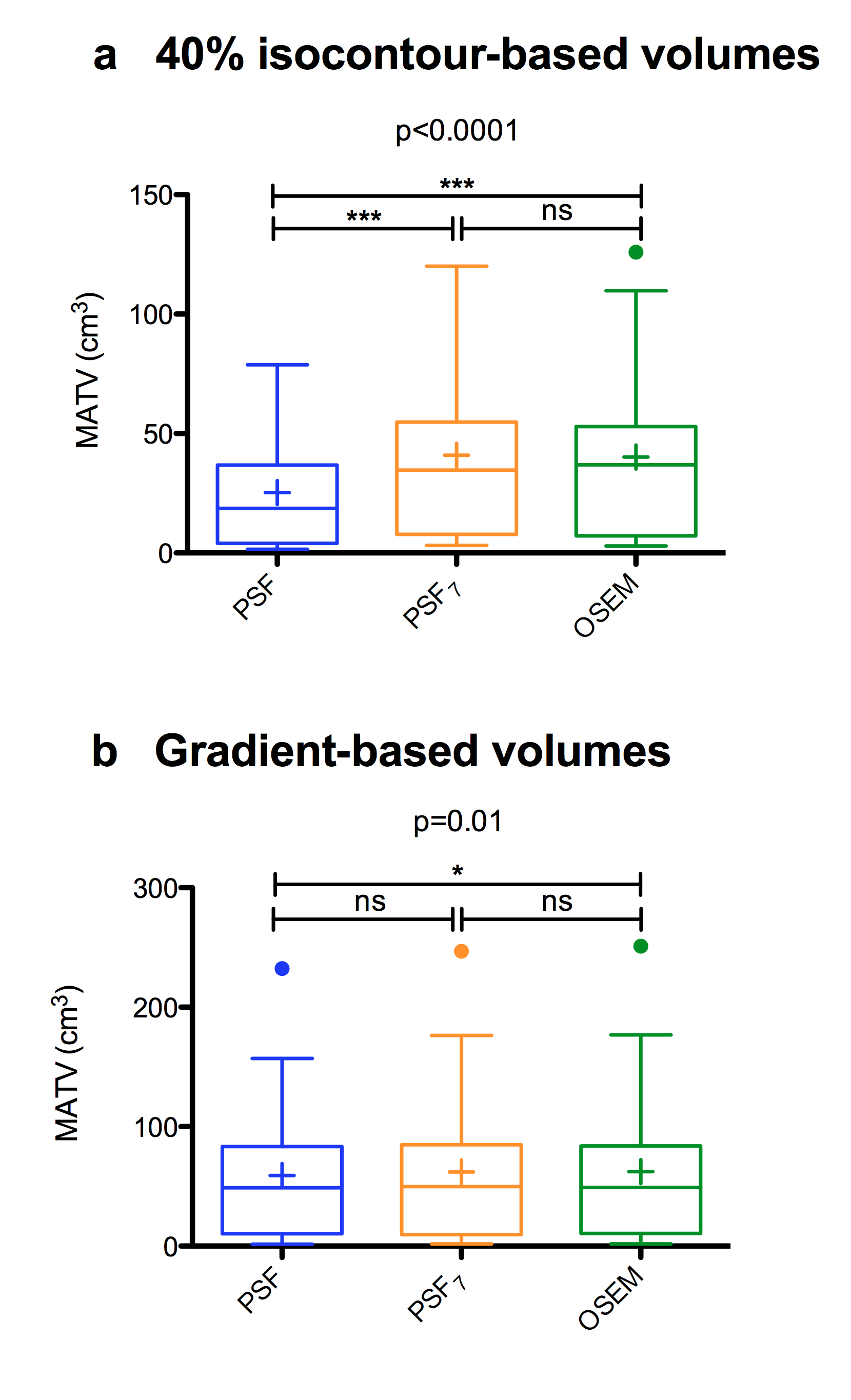

Supplement: Supplementary file 2 — Impact of the EARL harmonization strategy on MATVs defined by the isocontour and gradient-based delineation methods (observer 2). MATVs are shown as Tukey boxplots (lines displaying the median, 25th and 75th percentiles; cross represents the mean values). Legends for p values: ***<0.001; **<0.01; *<0.05. ns, not significant. (TIFF 17693 kb) [file 13550_2017_279_MOESM2_ESM.tiff]

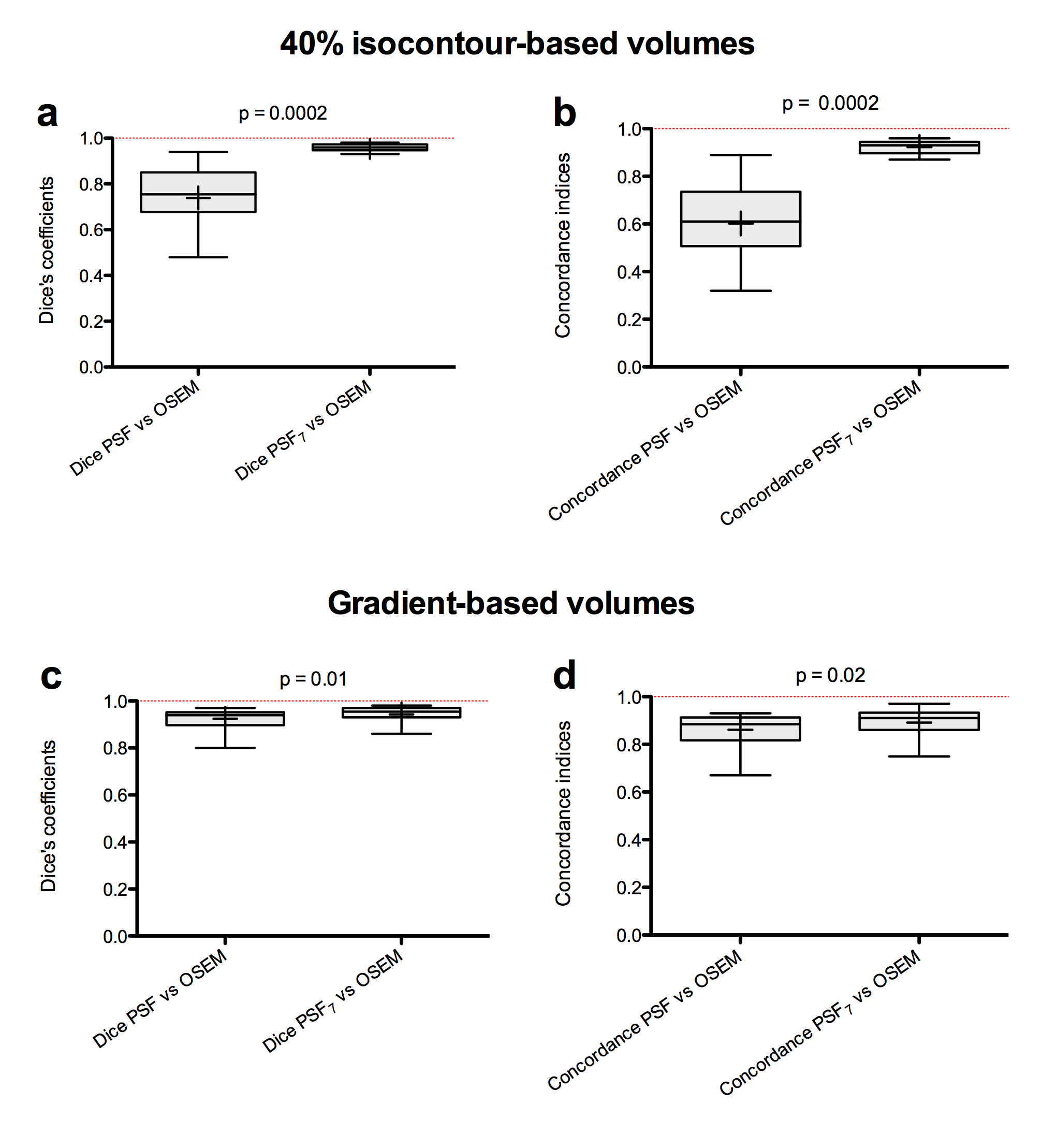

Supplement: Supplementary file 3 — Impact of the EARL harmonization strategy on Dice and concordance indices between MATVs extracted from OSEM images and PSF images (observer 2). The PSF7 images were filtered to meet EARL requirements while PSF images were optimised for diagnostic potential. Data are shown as Tukey boxplots (lines displaying the median, 25th, and 75th percentiles; crosses represents the mean values). Dice coefficients and concordance indices are shown for both the isocontour method (a and b) and gradient-based method (c and d). ns, not significant. (TIFF 17434 kb) [file 13550_2017_279_MOESM3_ESM.tiff]
